# An Unusual Amyloid Goiter in a 48-Year-Old Woman with Rheumatoid Arthritis, Secondary Amyloidosis and Renal Failure

**DOI:** 10.1155/2019/4291486

**Published:** 2019-12-28

**Authors:** B. López-Muñoz, L. Greco Bermúdez, D. Marín-Jiménez, M. F. Sánchez de la Fuente, A. Franca Capparelli, I. Mascarell Martínez, S. Serrano Corredor

**Affiliations:** ^1^Sección de Endocrinología y Nutrición, Hospital General Universitario de Alicante, ISABIAL-FISABIO, Valencia, Spain; ^2^Sección de Anatomía Patológica, Hospital General Universitario de Alicante, ISABIAL-FISABIO, Valencia, Spain; ^3^Sección de Anestesiología y Reanimación, Hospital General Universitario de Alicante, Alicante, Spain; ^4^Sección de Endocrinología y Nutrición, Hospital Vega Baja de Orihuela, Alicante, Spain

## Abstract

Amyloid goiter (AG) is characterized by the presence of deposits of amyloid protein in the thyroid tissue in sufficient amounts to produce enlargement of the gland, accompanied by fat deposition or thyrolipomatosis. It can be seen in long-standing inflammatory disorders, with the common characteristic of amyloidotic renal failure. In daily practice, practitioners should pay attention to the differential diagnosis in patients with suggestive co-morbidities for amyloidosis. The clinic is a progressive increase of the thyroid gland with compressive symptomatology (dyspnea, dysphagia, and changes in the voice). The main imaging finding is diffuse fatty infiltration of the thyroid. The amyloid goitre was most probably in the general context of amyloidosis, regardless of the other complications. We present a case of a 48-years-old female with amyloid goiter secondary to rheumatoid arthritis and renal failure.

## 1. Introduction

Amyloid goiter (AG) is characterized by the presence of deposits of amyloid protein in the thyroid tissue, accompanied by a diffuse accumulation of adipose tissue, capable of producing a significant enlargement of the gland [1]. Although the normal thyroid gland may have adipocytes in peri and subcapsular areas and near the vessels, and, sometimes, focal microscopic deposition of amyloid might be seen in both primary and secondary amyloidosis, amyloid goiter is a very rare entity with only a few cases described in the literature [1–3]. The main finding of this entity is the diffuse infiltration of fat from the thyroid gland, which is also known as thyroid lipomatosis or thyrolipomatosis (TL), a confusion factor in the terminology of this pathology.

## 2. Aim

We describe the case of a 48-years-old female with a very extensive diffuse fatty infiltration of the thyroid gland in an AG in the context of secondary amyloidosis.

## 3. Case Report

In July 2018, a 48-year-old woman was submitted in Endocrinology for goiter grade 2. She reported progressive dysphagia, with a feeling of “stop” the food bolus in the middle sternal area for 2-3 years.

She had a medical history of rheumatoid arthritis, diagnosed when she was 20 years old, in treatment with prednisone for 17 years continuously (current dose of 10 mg daily) and biological treatments (including rituximab and tocilizumab) at different times; secondary amyloidosis, diagnosed in 2010, by a rectal biopsy in a colonoscopy during the study of abdominal pain; colonic diverticula; and terminal renal failure, secondary to amyloidosis, in dialysis for 6 years and a half.

The patient was diagnosed of subclinical hyperthyroidism (TSH 0.21 mU/L (0.38–4.84), FT4 2.27 ng/dL (0.8–2.0) and FT3 2.38 *µ*g/ml (1.8–4.6)), without symptoms of thyroid hyperfunction and negative antithyroid antibodies. She was under treatment with Tiamazol 5 mg daily.

Thyroid ultrasound showed an enlargement thyroid gland, isoechoic and without nodules. Cervical computed tomography revealed an increase in size of thyroid gland, with a right thyroid lobe (LTD) of 5 × 4.7 × 8.8 cm, a left thyroid lobe (LTI) of 4 × 3.5 × 6.5 cm and an isthmus of 2.5 cm, with polylobulated contours. It displaced the carotid artery and the internal jugular vein laterally, without causing stenosis or displacement of the trachea. The thyroid parenchyma showed a marked decrease in density, similar to fat density. On T1- and T2-weighted images, hyperintense lobulated areas that corresponded to fatty attenuation areas on CT were noted. Core needle biopsy reported an intense adipose replacement of normal tissue and interstitial deposits of positive eosinophilic substance for Congo Red staining, with green birefringence in polarized light and positivity for Thioflavin with UV light, compatible with amyloid deposits.

**Figure 1:**
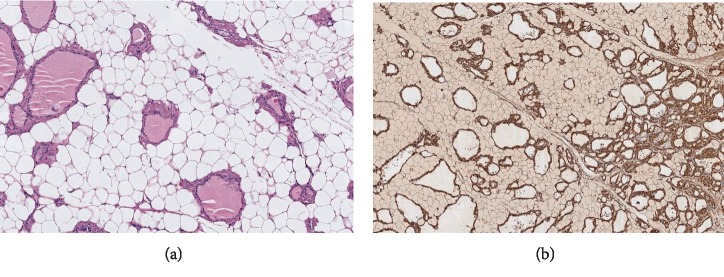
(a) (Hematosilin-Eosin) thyroid tissue replaced by adipocytes, preserving only the thyroid follicles, surrounded by eosinophilic, hyaline, and amorphous aggregates, (b) (amyloid A staining) are positive in immunohistochemistry to amyloid AA antibody.

With the diagnosis of amyloid goiter, total thyroidectomy was performed. During the surgery, a large thyroid mass was found. It seemed to be like a big, friable, lipoma, completely different from a normal thyroid gland. Three fragments of tissue with dimensions of 13.5 × 4.5, 6.5 × 3.5 and 3.5 × 2.5 cm, respectively, were referred to pathological anatomy. The appearance of the fragment was like adipose tissue, surrounded by a reddish margin that seemed to be the thyroid parenchyma. Histologically, a diffuse adipose metaplasia of the thyroid stroma was observed, associated with the deposition of amyloid material in the vascular walls and in the thyroid interstitium (Figure 1). The postoperative coursed without incidences.

## 4. Discussion

Amyloid goiter is defined by the presence of amyloid protein in the thyroid in sufficient amounts to produce enlargement of the gland accompanied by fat deposition of varying extents [1, 4]. It has been described associated in both primary and secondary amyloidosis with long-standing inflammatory disorders (familiar Mediterranean fever, rheumatoid arthritis, ankylopoietic espodilitis, chronic osteomyelitis, tuberculosis, bronchiectasis and other connective tissue diseases), and, more rarely, with neoplastic processes (medullary carcinoma of the thyroid), with the common characteristic of amyloidotic renal failure [2].

Its current incidence and prevalence are unknown. It has been described associated with as a primary amyloidosis in 0.04% and a familial Mediterranean fever in 0.27% [1].

References in literature on AG are limited. Until 2000, García-Villanueva et al. referred 200–250 published cases [5]. In our review, we have only found 9 cases published in the last 10 years, all of them were associated with kidney disease (CKD), with or without kidney transplant: 3 of them were associated with familial Mediterranean fever [1, 6], 2 cases were associated with rheumatoid arthritis [3, 7], 1 case was associated with bronchiectasis [2], another case with bronchiectasis and ankylosing spondylitis [1], and other 2 cases were associated with Crohn's disease [4] and multiple myeloma [8] (Table 1). Hijazi et al. [9] reviewed four more cases of thyrolipomatosis until 2018, publishing a new case in a 53-year-old woman with renal failure secondary to diabetic nephropathy, without anatomopathological study for amyloid.

The pathophysiology of diffuse proliferation of mature adipose tissue in the thyroid gland is unclear. Fat accumulation in amyloid goiters can be explained by tissue hypoxia secondary to progressive capillary and thyroid follicle destruction due to amyloid deposition. Tissue hypoxia may result in metaplasia of stromal fibroblasts to become adipose cells, which accumulate adipose tissue in the gland, increasing its size [1, 6–8].

Thyroid lipomatosis is clinically apparent due to a progressive enlargement of the thyroid that can involve the airway and/or upper gastrointestinal tract, producing dyspnea, dysphagia, and changes in the voice [7]. They tend to show rapid progression, which means that differential diagnosis must be made with malignant processes such as lymphoma or anaplastic cancer [6, 7].

Physical examination usually shows a soft, nontender goiter that is nodular or diffuse. In most cases, tests show normal thyroid function, but both hyperthyroidism and hypothyroidism have been described in a few patients, usually mild, like our case [7].

Imaging patterns in amyloid goiters may vary depending on the amount of fat and amyloid deposition. By ultrasonography, amyloid deposition has a hypoechoic pattern, with or without nodules. In our case, as fatty infiltration predominated, sonography pattern was isoechoic and diffuse. CT scan show an enlarged thyroid with well-defined limits and low density, in the range of adipose tissue (−10 to −50 Hounsfield Units). On MRI, fatty infiltration causes increased signal intensity both on T1- and T2-weighted images, with suppression on fat-saturated sequences. Cases with diffused fatty infiltration can be difficult to differentiate from thyrolipomatosis [7].

In a pathological examination, the thyroid reveals a loss of normal architecture with diffuse infiltration of mature adipose tissue without atypias. The number of follicles decreases and tends to be distorted and surrounded by an eosinophilic acellular amorphous material. There deposits are positive for Congo Red stained and for amyloid AA antibody in immunohistochemistry, showing green birefringence with polarized light. Some of the published cases have reported massively enlarged thyroids with masses of 500–700 g [7].

The usual treatment is total thyroidectomy [3], performed in our case and in the 9 cases mentioned, necessary both to relieve symptoms and to establish the definitive diagnosis. Pre-operatory suspicion is important if there are long-standing co-morbidities, including confirmed amyloidiosis, because it is useful to guide the surgeon, due to the risk of local extension and differential diagnosis with a thyroid carcinoma.

## 5. Conclusion

Amyloid goiter is a rare entity, which should be suspected in the presence of a goiter, in the context of a systemic disease associated with renal failure with/without transplantation. Systemic amyloidosis is usually accompanied by fatty deposits in the thyroid, but enlargement of the gland due to a thyrolipomatosis is infrequent. We believe that in literature there is not a good differentiation between the concepts of AG vs TL, with an overlap in the diagnostic criteria between both entities. It remains to be clarified if it is a single entity or, on the contrary, they are two pathologies with shared characteristics.

## Figures and Tables

**Table 1:** Amyloid goiter cases published since 2010.

Reference	Age (years old), sex	Associated pathology	Clinical features	Thyroid function	Thyroid autoantibodies
[1]	47, male	Ankylosing spondylitis	Incidental enlarged thyroid gland in thoracic TC	—	—
		Bronchiectasia			
		AA			
[1]	43, male	Familiar Mediterranean fever	Enlarged thyroid gland in physical examination	Normal	—
		AA			
[1]	34, female	Familiar Mediterranean fever	—	—	—
		AA			
[2]	30, male	Bronchiectasia	Dysphagia for solids	Normal	AntiTPO –
		Primary adrenal insufficiency due to amyloidosis			AntiTG –
[3]	52, female	Rheumatoid arthritis	Dyspnea	Normal	AntiTPO –
		AA	Neck swelling		AntiTG –
[4]	55, male	Crohn's disease	Neck swelling	Normal	—
		AA			
		Kidney transplant			
[6]	31, female	Familiar Mediterranean fever	Enlarged thyroid gland in physical examination	Normal	—
		AA			
[7]	36, female	Rheumatoid arthritis	Dyspnea	Normal	—
		AA	Enlarged thyroid gland in physical examination		
		Kidney transplant			
[8]	45, male	Multiple mieloma	Dysphagia	Normal	—
		AA	Dyspnea		

AA: secondary amyloidosis, TC: computer tomography, antiTPO: antimicrosomal antibodies, antiTG: antithyroglobulin antibodies.

## References

[1] Bakan S., Kandemirli S. G., Akbas S. (2017). Amyloid goiter: a diagnosis to consider in diffuse fatty infiltration of the thyroid. *Journal of Ultrasound in Medicine*.

[2] Oueslati I., Khiari K., Kaaroud H. (2016). Amyloid goiter as the first manifestation of systemic amyloidosis. *La Tunisie Médicale*.

[3] Uzum G., Kaya F. O., Uzum A. K. (2013). Amyloid goiter associated with amyloidosis secondary to rheumatoid arthritis. *Case Reports in Medicine*.

[4] Jacques T. A., Stearns M. P. (2013). Diffuse lipomatosis of the thyroid with amyloid deposition. *Journal of Laryngology & Otology*.

[5] García Villanueva A., García Villanueva M. J., García Villanueva M. (2013). Surgical considerations about amyloid goiter. *Endocrinology and Nutrition*.

[6] Aksu A. O., Ozmen M. N., Oguz K. K., Akinci D., Yasavun U., Firat P. (2010). Diffuse fatty infiltration of the thyroid gland in amyloidosis: sonographic, computed tomographic, and magnetic resonance imaging findings. *Journal of Ultrasound in Medicine*.

[7] Bell S., Sosa G. A., Del Valle Jaen A., Russo Picasso M. F. (2016). Thyroid lipomatosis in a 36-year-old patient with rheumatoid arthritis and a kidney transplant. *Endocrinology, Diabetes & Metabolism Case Reports*.

[8] Cannizzaro M. A., Bianco S. L., Saliba W. (2018). A rare case of primary thyroid amyloidosis. *International Journal of Surgery Case Reports*.

[9] Hijazi D. M., Addas F. A., Alghanmi N. M., Marzouki H. Z., Merdad M. A. (2018). An Enlarged goiter presenting with a rare diffuse lipomatosis of the thyroid gland. *American Journal of Case Reports*.

